# Proteomic Investigation of the Role of Nucleostemin in Nucleophosmin-Mutated OCI-AML 3 Cell Line

**DOI:** 10.3390/ijms23147655

**Published:** 2022-07-11

**Authors:** Ilaria Cela, Maria Concetta Cufaro, Maurine Fucito, Damiana Pieragostino, Paola Lanuti, Michele Sallese, Piero Del Boccio, Adele Di Matteo, Nerino Allocati, Vincenzo De Laurenzi, Luca Federici

**Affiliations:** 1 Department of Innovative Technologies in Medicine & Dentistry, University “G. d’Annunzio” of Chieti-Pescara, 66100 Chieti, Italy; ilaria.cela@unich.it (I.C.); maurine.fucito@unich.it (M.F.); damiana.pieragostino@unich.it (D.P.); michele.sallese@unich.it (M.S.); nerino.allocati@unich.it (N.A.); vincenzo.delaurenzi@unich.it (V.D.L.); 2 Center for Advanced Studies and Technology (CAST), University “G. d’Annunzio” of Chieti-Pescara, 66100 Chieti, Italy; maria.cufaro@unich.it (M.C.C.); paola.lanuti@unich.it (P.L.); piero.delboccio@unich.it (P.D.B.); 3 Department of Pharmacy, University “G. d’Annunzio” of Chieti-Pescara, 66100 Chieti, Italy; 4 Department of Medicine and Aging Science, University “G. d’Annunzio” of Chieti-Pescara, 66100 Chieti, Italy; 5 Institute of Molecular Biology and Pathology, National Research Council of Italy, P.le Aldo Moro 5, 00185 Rome, Italy; adele.dimatteo@cnr.it

**Keywords:** shotgun proteomics, nucleophosmin, acute myeloid leukaemia, nucleostemin

## Abstract

Nucleostemin (NS; a product of the *GNL3* gene) is a nucleolar–nucleoplasm shuttling GTPase whose levels are high in stem cells and rapidly decrease upon differentiation. NS levels are also high in several solid and hematological neoplasms, including acute myeloid leukaemia (AML). While a role in telomere maintenance, response to stress stimuli and favoring DNA repair has been proposed in solid cancers, little or no information is available as to the role of nucleostemin in AML. Here, we investigate this issue via a proteomics approach. We use as a model system the OCI-AML 3 cell line harboring a heterozygous mutation at the *NPM1* gene, which is the most frequent driver mutation in AML (approximately 30% of total AML cases). We show that NS is highly expressed in this cell line, and, contrary to what has previously been shown in other cancers, that its presence is dispensable for cell growth and viability. However, proteomics analysis of the OCI-AML 3 cell line before and after nucleostemin (NS) silencing showed several effects on different biological functions, as highlighted by ingenuity pathway analysis (IPA). In particular, we report an effect of down-regulating DNA repair through homologous recombination, and we confirmed a higher DNA damage rate in OCI-AML 3 cells when NS is depleted, which considerably increases upon stress induced by the topoisomerase II inhibitor etoposide. The data used are available via ProteomeXchange with the identifier PXD034012.

## 1. Introduction

Nucleostemin (NS; a product of the *GNL3* gene) is a nucleolar–nucleoplasm shuttling protein, mainly resident in nucleoli, which belongs to the Ylqf/YawG family of circularly permutated GTPases. NS levels are high in stem cells, while its expression rapidly decreases upon differentiation [1]. NS is involved in several different processes regarding embryonic development, pluripotency reprogramming, telomere maintenance and tissue regeneration [2,3,4,5,6,7]. While generally absent in normal tissues, NS is abundantly expressed in different solid tumours. For instance, NS was shown to be necessary for mammary tumour progression [8]. Indeed, mammary tumour cells were shown to be susceptible to DNA damage upon NS silencing and, conversely, to acquire protection from hydroxyurea treatment upon NS overexpression [8]. In hepatocellular carcinoma, NS expression was more abundant in high-grade and metastatic forms and was a predictor of shorter overall and progression-free survival [6]. Also in this case, NS overexpression was shown to protect cancer cells from drug-induced DNA damage, while its downregulation was shown to increase cytosolic double-stranded DNA, while upregulating PD-L1 and cytokine expression [6]. In glioma, a positive correlation between NS upregulation and cell proliferation was found, while NS downregulation alleviated this phenotype by reducing β-Catenin transportation into the nucleus [9]. Finally, in oral squamous cell carcinoma, overexpression of NS was again shown to increase the proliferation and invasive potential of cancer cells, while its downregulation suppressed the invasiveness of cells [10].

Mechanistically, most NS functions are performed in the nucleoplasm, and it was initially suggested that the GTP/GDP binding state of the protein modulates its nucleolus–nucleoplasm shuttling, possibly through variations in inter-domain contacts [11]. Later, a role in telomere maintenance was demonstrated through NS binding of the telomeric-repeat binding factor 1 (TRF1) which inhibits TRF1 association to the telomere and promotes its degradation [12,13]. Subsequently, it was shown that when NS moves to the nucleoplasm, because of nucleolar disassembly during cell cycle or because of nucleolar stress, it interacts with MDM2 and competes with L23 for MDM2 binding, while NS depletion decreases MDM2 levels and triggers G2/M arrest. Importantly, neural-specific knockout of NS in mice was shown to trigger spontaneous DNA damage in embryos, promoting growth arrest independent of p53 status or rRNA. Indeed, NS was shown to participate in DNA repair through homologous recombination by means of its direct recruitment to DNA damage sites where, in turn, it helps the recruitment of RAD51 [13].

The involvement of NS in promoting homologous recombination appears to be particularly relevant to the understanding of its role in cancer. Indeed, the increased expression of NS in multiple neoplasms suggests that it might act as an oncogene product. On the other hand, its involvement in DNA repair is more reminiscent of the behaviour of a tumour suppressor, since homologous recombination deficiency is a known cancer-predisposing factor. To remove this contradiction, it has been suggested [6,8] that differences in DNA damage and repair pathways might be taken into consideration. In particular, impaired or compromised DNA repair through homologous recombination might be detrimental to already transformed tumours, which are mitotically very active, because it may predispose them to excessive DNA damage that might lead to mitotic catastrophe. In light of this, reinforced NS expression may serve to safeguard genome integrity in highly mitotically active cells [6,8].

A role for NS has been highlighted also in haematological malignancies. It has been shown that NS is expressed in the chronic myelogenous leukaemia cell line K562 and that its depletion induces post-G1 arrest apoptosis [14]. NS knockdown in T-cell acute lymphoblastic leukaemia MOLT-4 was shown to trigger cell cycle arrest and apoptosis via p53 activation [15]. In the acute promyelocytic leukaemia cell line NB4, NS silencing was shown to induce differentiation and potentiate all-trans-retinoic acid-mediated cell death [16]. NS expression was also detected in the p53-null acute myeloid leukaemia (AML) HL-60 cell line; also, in this case NS silencing was associated with apoptosis, possibly through activation of the JNK pathway [17]. Importantly, transcript levels of NS were analysed in the bone marrow of newly diagnosed AML patients; they were found to be higher as compared to normal bone marrow in all FAB subtypes and to correlate with blast percentages [18].

AML is a heterogeneous disease caused by a limited subset of chromosomal translocations or mutations in selected genes. Among them, the gene coding for nucleophosmin (NPM1) is the most frequently mutated, accounting for approximately 30% of all AML cases and 60% of those with normal karyotype. Mutations drive the destabilization or total unfolding of the protein C-terminal domain [19,20,21], with the loss of nucleolar retention and the aberrant and stable localization of the mutated protein in the cytosol (hence its being called NPM1c+, from cytoplasmic positive) [22,23]. Interestingly, NS and NPM1 were shown to be interacting partners [24], even though the role of this interaction has not yet been fully elucidated.

While a good mechanistic insight has been gained into the role of NS in different solid tumours, as highlighted before, the same cannot be said for haematological cancers. In particular, NS has never been investigated before in the context of AML with NPM1 mutation.

Here, we address this issue by taking as a model system the OCI-AML 3 cell line, which bears the most common heterozygous NPM1 mutation [25]. We assessed a strong expression of NS in OCI-AML 3 cells, even though we observed that its presence is dispensable for cell growth and viability. Therefore, we performed a comprehensive analysis of this cell line proteome before and after NS silencing. This, to the best of our knowledge, is the first proteomics investigation of OCI-AML 3 cells in particular and of AML with NPM1c+ more generally. Data were analysed using Ingenuity Pathway Analysis (IPA software). Among the biological functions that were suggested to be affected by the proteomics analysis, we focussed our attention on the effect of NS silencing in down-regulating DNA repair through homologous recombination. Indeed, we observed a higher DNA damage rate with respect to the control and this effect was further increased upon treatments with etoposide. Overall, our data suggest, similarly to what observed in solid cancers, a role for NS in counteracting excessive DNA damage under stress conditions, which might be deleterious for the mitosis of rapidly proliferating AML cells with NPM1 mutations.

## 2. Results

### 2.1. Analysis of NS Silencing in OCI-AML 3 Cells

In order to investigate the role of NS in AML with NPM1 mutation, we used as a model system the OCI-AML 3 cell line bearing the most common NPM1 mutation, i.e., type A. Preliminarily, we assessed basal NS expression in a panel of AML cell lines for comparison (Figure S1). We found that NS was expressed in all tested cell lines, albeit at different levels.

**Figure 1:**
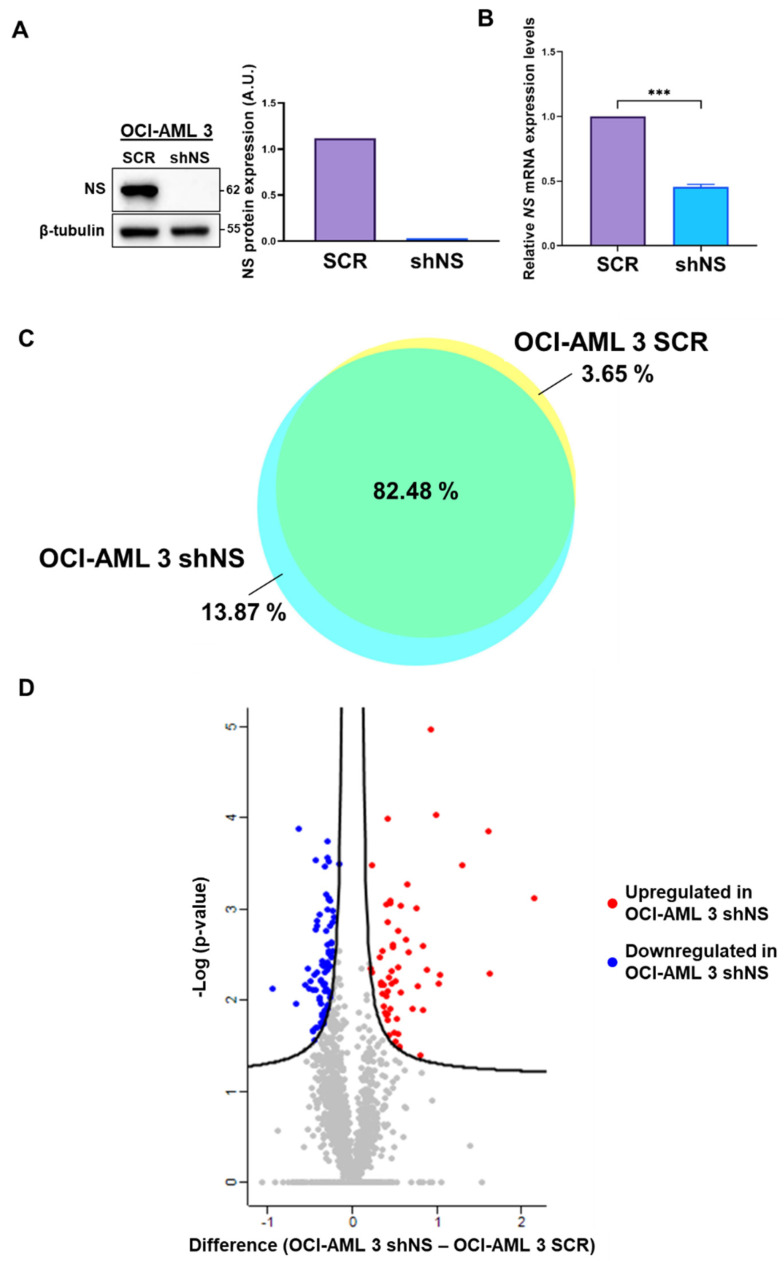
NS expression, silencing and differential proteomics analysis of OCI-AML 3 cells. (**A**) Western blot (WB) images of NS protein levels in OCI-AML 3 cells in SCR (not silenced) and shNS (silenced for NS) after 72 h of doxycycline induction. Twenty-five micrograms of total lysates were loaded for each sample. Histogram represents intensity levels of protein bands of Western blot image normalized for β-tubulin protein expression. (**B**) NS mRNA expression levels in control (SCR) and silenced (shNS) OCI-AML 3 cells after 72 h of doxycycline treatment. mRNA levels were evaluated by qRT-PCR and normalized to B2M expression levels. Asterisks represent significant differences with respect to SCR control. *** *p* < 0.001, Student’s *t*-test. (**C**) The Venn diagram highlights the commonly expressed proteins between NS-silenced (shNS) or not (SCR) OCI-AML 3 cells. (**D**) Volcano plot of common proteins graphed by difference parameter and –Log(*p*-value). Blue dots represent proteins that were significantly down-regulated in OCI-AML 3 shNS, while red dots indicate proteins that were significantly up-regulated in OCI-AML 3 shNS compared to the control OCI-AML 3 SCR. Grey dots represent proteins that were not differentially expressed in the comparison carried out.

Knockdown of NS was performed using a stable and inducible silencing system expressing a short-hairpin RNA (shRNA) specifically targeting NS mRNA (shNS), whose expression is induced only in the presence of doxycycline. First of all, we observed a strong expression of NS in OCI-AML 3 SCR control cells, which was markedly reduced after 72 h of doxycycline treatment. Under this condition, NS protein levels were less than 10% in shNS OCI-AML 3 cells in comparison with the SCR control (Figure 1A). In addition, mRNA expression levels of NS in silenced cells were significantly reduced—by at least of 50%—as compared to the SCR control (Figure 1B). We then performed cell proliferation assays under NS silencing (shNS) or not (SCR) conditions, by considering 72 h doxycycline induction as the starting point for the indicated time points. However, as reported in Supplementary Figure S2, and, contrary to what was assessed for other cell lines of different histological origin, no significant differences were observed in the proliferation of NS-silenced cells in comparison with SCR control cells.

### 2.2. Comparative Proteomics Analysis of OCI-AML 3 Cell Lines

To further shed light on the role of NS expression in OCI-AML 3 cells, we performed shotgun proteomics analysis of shNS cells and their SCR control. We could identify and quantify 1298 proteins for OCI-AML 3 SCR and 1452 proteins for OCI-AML 3 shNS. Among them, 1245 proteins were in common between the two conditions, as reported in the Venn diagram of Figure 1C, consisting of 82.5% of all identified proteins. The whole list of the quantified proteins is reported in Supplementary Table S1, sheet “quantification”. Moreover, a high Pearson correlation was found between the two OCI-AML 3 cell lines, as shown in the density plot of Supplementary Figure S3, with a Pearson correlation coefficient of 0.99. Among all the common proteins analyzed, 133 significant differentially expressed proteins (*p*-value < 0.05) were determined via *t*-test. In particular, in Figure 1D common proteins are graphed in a volcano plot showing that 76 and 57 proteins were, respectively, significantly down-expressed (blue dots) and over-expressed (red dots) in OCI-AML 3 shNS compared to the SCR control. All differential proteins shown in the volcano plot are listed in Table S1, sheet “differential proteins”.

### 2.3. Functional Proteomics Analysis

Quantitative proteomics data obtained with MaxQuant software were used for functional GO reclassification analysis. The protein ratio (OCI-AML 3 shNS/OCI-AML 3 SCR) was subjected to “Core Analysis” through the Ingenuity Pathway Analysis (IPA) bioinformatics tool. The most relevant downstream biological processes that we found are reported in Table 1 with their corresponding z-score values. OCI-AML 3 cells with silenced NS significantly triggered biological processes related to down-regulation of cell movement, cell viability and homologous recombination as well as significantly up-regulated mechanisms linked to cell death of cancer cells and bone marrow lesions. The downregulation of cell viability and upregulation of cell death of cancer cells, as biological functions, however, were not immediately reflected in a loss of proliferation of NS-silenced cells, as already reported in Figure S2.

Moreover, NS silencing in OCI-AML 3 resulted in the up-regulation, although not in a significant way, of downstream functions associated with protein translation, degranulation of cells and DNA damage (see Supplementary Table S2). We then performed an upstream regulator analysis (URA), again using the IPA software. This analysis suggested many transcription factors or other protein actors probably related to the previously discussed biofunctions and, in addition, to cell cycle control, cell proliferation, differentiation and responses to cell stresses. The most significant upstream regulators are listed in Table 2 with their z-score activation values.

### 2.4. Nucleostemin Silencing in the NPM1-Mutated OCI-AML 3 Cell Line Affects the Pathways Involved in Homologous Recombination

**Figure 2:**
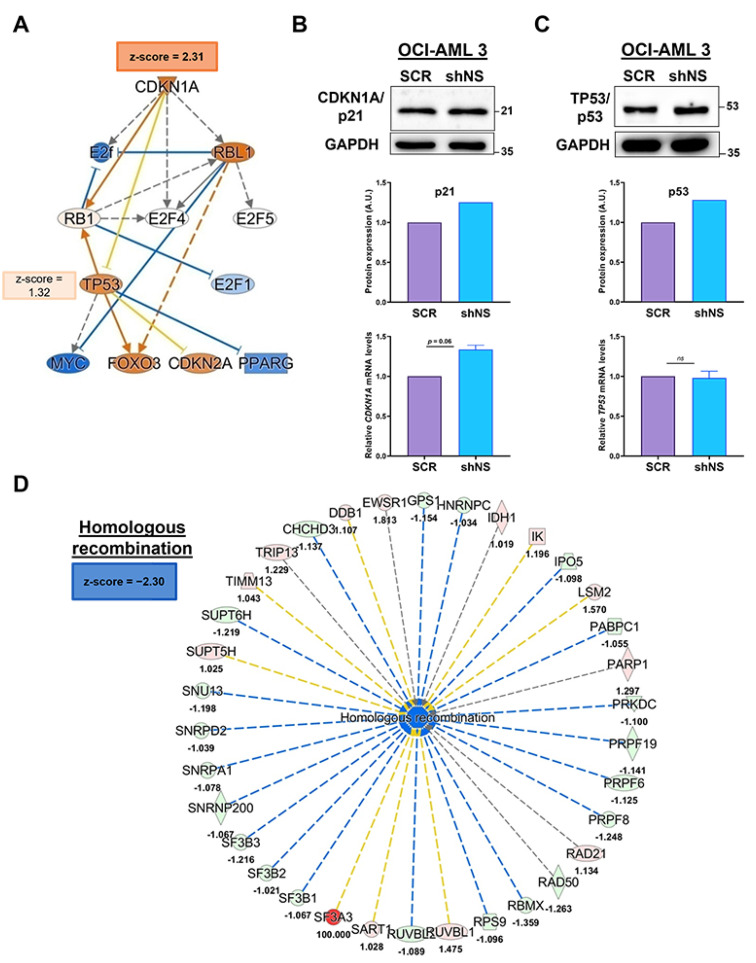
Functional Proteomics Analysis revealed the activation of CDKN1A/p21 as an upstream regulator in NS-silenced OCI-AML 3 cells corroborated with a strong inhibition of homologous recombination function. (**A**) Mechanistic network of the putative upstream regulator CDKN1A/p21 protein and its significant levels of activation predicted by IPA (z-score = 2.31) for the comparison of OCI-AML 3 shNS/OCI-AML 3 SCR. Within the network, some of the protein actors were directly or indirectly regulated by CDKN1A/p21, such as the TP53/p53 protein (z-score = 1.32, *not significant*), colored according to IPA color code (see Supplementary Figure S4), based on their predicted levels of activation/inhibition. WB images and their corresponding quantifications of protein expression levels of (**B**) CDKN1A/p21 and (**C**) TP53/p53 in NS-silenced (shNS) or not (SCR) OCI-AML 3 cells. For each lane, 25 µg of total lysates were loaded. GAPDH is reported as loading control. For each protein considered, mRNA expression levels are expressed as ratios over OCI-AML 3 SCR cells and normalized for B2M. (**D**) Downstream network analysis shows a strong inhibition of “*homologous recombination*” in OCI-AML 3 shNS compared to its SCR control (z-score = −2.30).

Among the most significantly modulated upstream regulators in the comparison of OCI-AML 3 shNS/OCI-AML 3 SCR (see Table 2), we focused our attention on cyclin-dependent kinase inhibitor 1 (CDKN1A/p21), which was significantly activated in OCI-AML 3 shNS cells, with a z-score of 2.31 (Figure 2A). The tumour suppressor CDKN1A/p21 plays a key role in cell cycle control and its levels are increased often through p53-mediated regulation after various cell stresses, including DNA damage [26,27]. To better highlight the role of p21 as a potential upstream regulator in NS-silenced OCI-AML 3, we show in Figure 2A its mechanistic network obtained by IPA which illustrates some protein actors directly modulated by p21 itself. This is the case, for example, for the cell cycle control-related family of transcription factors E2f and, among them, *E2F1* was predicted to be slightly inhibited, while the retinoblastoma-associated protein RB1 was predicted to be directly activated by CDKN1A/p21 (z-score = 2.12). Moreover, from the IPA analysis, the tumour suppressor p53 (TP53) was also predicted to be activated, though not in a significant manner (z-score = 1.32) (Figure 2A). We then evaluated both protein and transcriptional expression levels of CDKN1A/p21 in OCI-AML 3 SCR and shNS and we observed modestly increased levels of CDKN1A/p21 under NS silencing, thus confirming IPA predictions (Figure 2B). Given the direct link between CDKN1A/p21 and p53, we tested p53 protein and mRNA levels under NS depletion (shNS) or not (SCR) and we found slightly increased protein levels of p53, while its transcriptional levels were found to be unchanged (Figure 2C).

The modulation of CDKN1A/p21 upstream in the comparison of OCI-AML 3 shNS/OCI-AML 3 SCR cells is likely linked to cell cycle control and DNA damage. Indeed, the significant up-regulation of CDKN1A/p21 in OCI-AML 3 shNS was corroborated by the down-regulation of the “*homologous recombination*” biological function (z-score = −2.30; Figure 2D and Table 1) in the same cell line. This suggests that NS silencing in OCI-AML 3 cells might correlate with an increased DNA damage rate. Of note, NPM1, too, is known to be involved in DNA repair mechanisms [28,29], while recent evidence has shown the implication of NS in the homologous recombination pathway [6,8,30].

**Figure 3:**
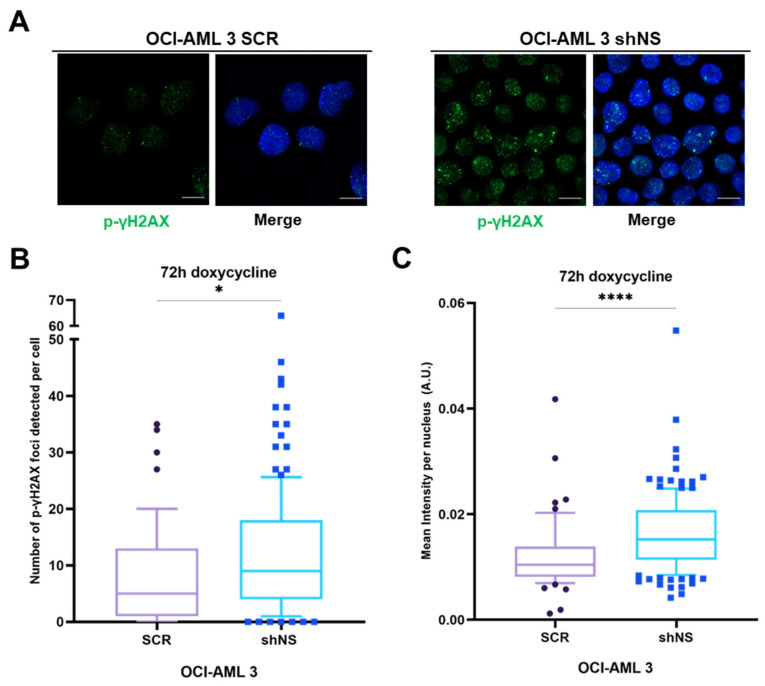
NS silencing in OCI-AML 3 cell lines induces higher DNA damage rates. (**A**) Representative immunofluorescence images (scale bar = 10 µm) of OCI-AML 3 cells not silenced (SCR) and silenced (shNS) for NS protein after 72 h induction with doxycycline. Cells were stained with anti-p-γH2AX antibody (green) to evaluate DNA damage foci within cell nuclei. Merge images are reported for each figure in which, beside anti-p-γH2AX foci, Hoechst 33342 (blue) staining corresponds to cell nuclei. (**B**) Distribution of the number of foci detected per each single cell analyzed with FoCo software from 10 images for, respectively, SCR and shNS OCI-AML 3. Medians for each distribution are highlighted in the box plot. * *p* < 0.05, non-parametric Student’s *t*-test. (**C**) Distribution of mean intensity per nucleus per each single cell measured for each condition of OCI-AML 3 cell lines after 72 h of doxycycline treatment by using FoCo software. Medians for each distribution are highlighted in the box plot. **** *p* < 0.0001, non-parametric Student’s *t*-test.

Homologous recombination is a DNA repair pathway also linked to cell cycle progression [31] and, given the fact that we found this pathway significantly down-regulated in NS-silenced OCI-AML 3 cells, we next wanted to investigate whether NS silencing resulted in increased DNA damage through the evaluation of the content of DNA damage foci (Figure 3). Phosphorylation of the histone γH2AX (p-γH2AX) is considered an early marker of DNA damage foci within nuclei and its presence functions as a signal for further recruitment of protein actors belonging to different DNA repair machineries, including the homologous recombination pathway [32]. To evaluate the presence of p-γH2AX-related foci, immunofluorescence images were taken after 72 h of doxycycline treatment in both SCR and shNS OCI-AML 3 cells. As shown in Figure 3A, p-γH2AX foci were more abundant in silenced cells in comparison to their SCR controls. In addition, p-γH2AX foci were quantified using FoCo software [33], thus obtaining the number of p-γH2AX foci per each single cell analysed, reported as a distribution in Figure 3B. The median number of γH2AX foci detected per single cell was found to be significantly higher in OCI-AML 3 shNS in comparison with control OCI-AML 3 SCR cells. Furthermore, as shown in Figure 3C, the distribution of intensities of overall p-γH2AX foci detected was significantly wider in NS-silenced OCI-AML 3 cells than the distribution of intensities detected in non-silenced OCI-AML 3 cells, with an intensity median value significantly increased in OCI-AML 3 shNS as further confirmation of the presence of more intense p-γH2AX foci within silenced cells in comparison with their non-silenced controls. All these observations suggest that silencing of NS is associated with a greater amount of DNA damage, which likely arises from diminished DNA repair. This could, in turn, be related to the down-regulation of the homologous recombination pathway that we found in these cells with respect to SCR controls through proteomic analysis (Table 1, Figure 2D).

### 2.5. Nucleostemin-Silenced Cells Are More Sensitive to DNA Damage Induced by Etoposide Treatment

**Figure 4:**
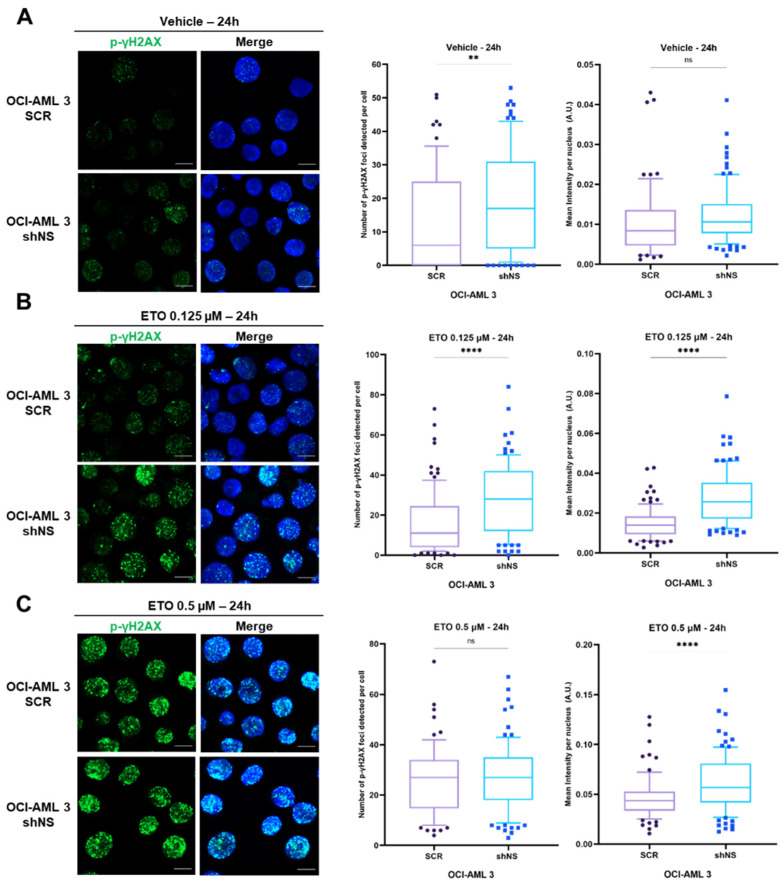
Etoposide treatments together with NS silencing increase DNA damage foci in OCI-AML 3 cell lines. Representative immunofluorescence images (scale bar = 10 µm) of OCI-AML 3 cells not silenced (SCR) and silenced (shNS) for NS protein under doxycycline induction after 24 h of DMSO 0.1% (vehicle) (**A**), 0.125 µM (**B**) or 0.5 µM etoposide (**C**) (ETO) treatments. Cells were stained with anti-p-γH2AX antibody (green dots) to evaluate DNA damage foci within cell nuclei. Merge images are reported for each figure in which, beside anti-p-γH2AX foci, Hoechst 33342 (blue) staining corresponds to cell nuclei. For each condition considered, the distribution of the number of foci detected per single cell (left) and the distribution of mean intensity per nucleus per cell (right) were analyzed with FoCo software (10 images taken) for, respectively, SCR and shNS OCI-AML 3 cells after 24 h of DMSO 0.1% (vehicle) (**A**), 0.125 µM (**B**) or 0.5 µM ETO (**C**) treatments. In both box plots represented, medians for each distribution are highlighted. ns, not significant; ** *p* < 0.01; **** *p* < 0.0001, non-parametric Student’s *t*-test.

To better understand to what extent the homologous recombination repair pathway is affected by NS depletion, we decided to treat both SCR and shNS cells with different concentrations of etoposide (ETO), which is known to elicit DNA damage by inducing double-strand breaks (DSBs) through the inhibition of topoisomerase II [34] (Figure 4). We first treated cells with vehicle to evaluate the putative effects of DMSO on foci number. As expected, we observed a significantly higher median value in the number of foci detected per cell in vehicle-treated OCI-AML 3 shNS, while distributions of overall intensity values per nucleus were comparable in both shNS and SCR OCI-AML 3 cells (Figure 4A). Then, cells were treated for 24 h with two different concentrations of etoposide (ETO; 0.125 µM and 0.5 µM) in order to induce substantial DNA damage without, however, leading to massive cell death. Upon treatment with 0.125 µM etoposide, NS-silenced OCI-AML 3 cells suffered a significantly higher DNA damage rate in comparison with their SCR control treated in the same way, as reported in Figure 4B. Moreover, upon treatment with the higher dose of 0.5 µM etoposide, although the distributions of number of foci detected were comparable, overall signal intensities of foci per nucleus were higher in NS-depleted OCI-AML 3 cells with respect to their SCR controls, thus suggesting wider areas where DNA is particularly damaged (Figure 4C). Collectively, these data suggest a higher sensitivity to etoposide in NS-depleted cells (Figure 4B).

**Figure 5:**
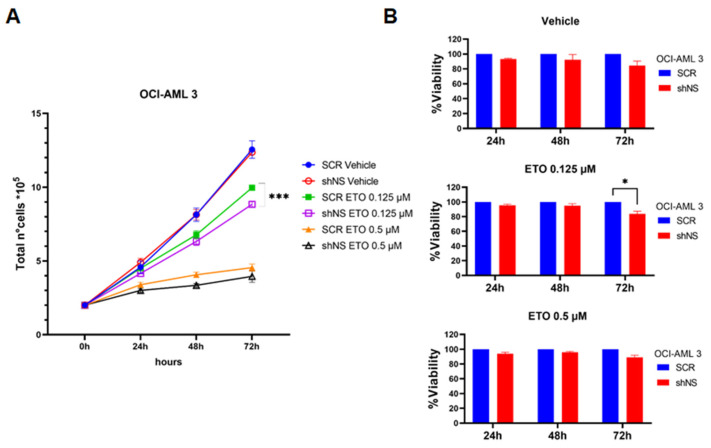
Lower doses of etoposide in combination with NS silencing reduce proliferation rates of OCI-AML 3 cells. (**A**) Proliferation rates of OCI-AML 3 cell lines, silenced (shNS) or not (SCR) for NS, and treated with DMSO 0.1% (vehicle), 0.125 µM or 0.5 µM etoposide (ETO), graphed on the base of the total number of cells counted at each time point for each condition considered, as indicated. Seventy-two hours post-induction with doxycycline was considered the starting point of the curve. *** *p* < 0.001, Student’s *t*-test. Representative data graphed as means ± SDs of two independent experiments, each performed in quadruplicate. (**B**) Cell viability assays of OCI-AML 3 SCR and shNS cells treated with DMSO 0.1% (vehicle), 0.125 µM or 0.5 µM etoposide (ETO), expressed as the percentage fold change ratio of viability of NS-silenced OCI-AML 3 cells over SCR controls for each time point indicated and for each treated condition, considering 72 h doxycycline post-induction as the starting point of the curve. * *p* < 0.05, two-way ANOVA. Representative data graphed as means ± SDs of three independent experiments, each performed in eight technical replicates.

In order to evaluate whether impaired homologous recombination in NS-silenced OCI-AML 3 cells resulted in altered proliferation and cell viability under stress conditions due to higher induced DNA damage, we treated both OCI-AML 3 SCR and OCI-AML 3 shNS cells with the same concentrations of etoposide used for immunofluorescence assays to evaluate their ability to proliferate in the presence of this DNA-damage-inducing agent. After confirming that proliferation of NS-silenced OCI-AML 3 cells was unchanged in comparison with SCR control cells in unstressed conditions (Figure 5A), consistently with previous data (Figure S2), we observed that the higher dose of etoposide (0.5 µM) exerted a massive and comparable anti-proliferative effect on both NS-silenced and expressing OCI-AML 3 cells (Figure 5A). Conversely, OCI-AML 3 shNS cells treated with the lower etoposide dose of 0.125 µM displayed significantly reduced cell growth compared to control cells after 72 h of treatment (Figure 5A). This was accompanied by a very modest but significant effect on cell viability at the same lower ETO dose (Figure 5B).

## 3. Discussion

NS is a relatively recently discovered protein whose multifaceted properties are yet to be fully disclosed. Given its abundant expression in stem cells, it is frequently used as a marker of stemness in laboratory practice. However, while its levels rapidly decrease in differentiated cells, they remain high in multiple cancers [1,6,8,9,10]. This is also the case with AML, and we have confirmed here that NS is abundantly expressed in the NPM1-mutated cell line OCI-AML 3. Over the years, many different roles in telomere maintenance, cell cycle control and DNA repair have been proposed in several solid cancers, while mechanistic insight into the role of NS in AML is largely lacking [6,7,8,35,36,37]. Here, we have started to address this issue by taking into consideration a particular subtype of AML, i.e., the one with heterozygous NPM1 mutation [20,21,22,23]. We found that NS is dispensable for OCI-AML 3 cell growth and viability in normal conditions. To gain further insight, we performed shotgun proteomic analyses of our cells before and after NS depletion, thus investigating for the first time the overall proteome of an AML cell line with NPM1 mutation and revealing that consistent changes are detected when NS is depleted (see Figure 1). We analyzed our quantitative data with the aid of IPA software and found significantly altered biological functions (see Table 1). Among them, we found significant upregulation of cell death and bone marrow lesions in NS-silenced cells with respect to the control, suggestive of a protective role for NS. This was corroborated by the biological functions that were found to be significantly inhibited, mostly related to DNA repair through homologous recombination and cell survival. The IPA software also allowed us to predict the upstream regulators, i.e., those transcription factors or protein actors, whose altered levels may be partly responsible for the above-mentioned alterations in biological functions. We found several significantly altered upstream regulators (see Table 2) upon NS silencing, which, taken together, suggest the implication of NS in pathways related to cellular responses to stress and the regulation of cell death. Among the altered upstream regulators, to gain a functional confirmation of our proteomics data, we further investigated the upstream *CDKN1A*/*p21* that was predicted to be significantly upregulated. Indeed, p21 was found to be increased in NS-silenced cells, both at the protein and mRNA levels, albeit only modestly. This was also reflected in a modest increase in p53 levels, also predicted by the upstream regulators analysis, although not significantly. These variations might reflect a putative role of NS in protecting rapidly proliferating OCI-AML 3 cells from excessive DNA damage by enhancing their DNA-repair capabilities. This hypothesis would be in line with what was previously observed in other cancers [6,8,30]; however, experimental confirmation is needed. Therefore, we evaluated the levels of DNA damage foci in both conditions and found them to be significantly increased in NS-silenced cells, both in terms of number and median intensity. This difference may be explained via our proteomics analysis as being dependent on compromised DNA repair through homologous recombination and may not be detrimental to cell viability in normal conditions given the presence of alternative DNA repair mechanisms. However, in conditions of stress that increase DNA damage, the protective role of NS might emerge more clearly. To test this hypothesis, we repeated our experiments by treating cells with etoposide, a topoisomerase II inhibitor that is known to elicit DNA double strand breaks. Importantly, the increase in the number and intensity of foci, already higher in NS-silenced cells in the absence of stressors, was found to be exacerbated under etoposide treatment, also at the lower dosage of 0.125 μM, which is only modestly toxic for OCI-AML 3 cells (see Figure 5). Indeed, shNS OCI-AML 3 cells were found to be more susceptible to low doses of etoposide both in terms of cell growth and viability.

In conclusion, in this work we have started to investigate the role of NS in AML with NPM1 mutation, the most common AML subtype, taking advantage of a proteomic approach. Our data are in line with what has already been shown for other solid tumours, including recent work on breast [8], hepatocellular [6] and ovarian cancer [30], and suggest the involvement of NS in different pathways, though most prominently in DNA repair through homologous recombination. These findings highlight a role that is not immediately or solely recognizable as that of an oncogene, as NS overexpression would suggest, or as a tumour suppressor, as its involvement in sustaining DNA repair would also suggest. Rather, our findings support and extend to AML a previous hypothesis [6,8] concerning a protective role for this protein in already transformed cells that need, while progressing over time and accumulating mutations, to avoid excessive DNA damage. Indeed, in the stressed tumoural micro-environment, rapidly proliferating cancer cells may need the presence of compensatory mechanisms to avoid excessive genomic instability and consequent mitotic catastrophe. Given this perspective, it is not surprising that NS has been suggested as a putative drug target in different solid tumours. As to AML, this is a tumour that is currently treated with high-dose chemotherapy (the so-called 7 + 3 regimen) followed, depending on the tumour genotype, by bone marrow transplantation. Remission is almost always achieved but, unfortunately, relapse is just as frequent. Therefore, novel therapeutic options to be used in combination with those already existent are needed. Our data on etoposide treatment allows us to hypothesize that inhibition of NS might be synergic with standard chemotherapy and improve AML prognosis. However, a great deal of research is still necessary to reach this goal. Indeed, we still need to clarify what to inhibit in NS under a therapeutic perspective and how to go about it. Our study and those of others suggest that the recruitment of NS to DNA damage sites or its interaction with key players in DNA repair through homologous recombination may be targeted. We suggest that future research on NS may be partly directed towards investigating these interactions, also from a structural biological point of view.

## 4. Methods and Materials

### 4.1. Cell Cultures and Reagents

All AML cell lines used were grown in suspension. OCI-AML 3 and 2 were grown in alpha-MEM (Lonza, Basel, Switzerland; #BE02-002F) supplemented with 20% (*v*/*v*) fetal serum bovine (FBS) (Gibco, Waltham, MA, USA); HL-60 cells were grown in Iscove’s modified Dulbecco’s medium (Euroclone, Pero (MI), Italy; #ECB2072) supplemented with 20% (*v*/*v*) of FBS (Gibco, Waltham, MA, USA); THP-1 cells were grown in RPMI (Gibco, Waltham, MA, USA; #11875093) supplemented with 10% (*v*/*v*) FBS (Gibco, Waltham, MA USA). HEK 293T were grown in DMEM (Gibco, Waltham, MA, USA; #41966029) supplemented with 10% (*v*/*v*) FBS (Gibco, Waltham, MA, USA) and penicillin–streptomycin 10,000 U/mL (Gibco, Waltham, MA, USA). All the cell lines were grown at 37 °C in a humidified atmosphere of 5% (*v*/*v*) CO_2_. Etoposide (VP-16; Sellek Chemicals, Houston, TX, USA) was resuspended in DMSO (Sigma-Aldrich, St. Louis, MI, USA) as stock solution at 500 µM.

### 4.2. Lentiviral Infections and Silencing of Nucleostemin Protein in OCI-AML 3 Cells

Lentiviruses were produced by transient co-transfection of packaging cell line HEK 293T with a three-plasmid expression system using a calcium phosphate transfection kit (Invitrogen, Thermo-Fisher, Waltham, MA, USA; #K278001). Lentiviral plasmids pTRIPZ non-silencing (SCR) and sh*GNL3* (shNS) (clone ID: V2THS_251446) were purchased from Horizon. pTRIPZ plasmid inducible co-express short-hairpin RNAs together with TurboRFP protein were used to monitor levels of induction under doxycycline treatment. 

HEK 293T cells were incubated for 6 h with transfection reagents. Lentiviral particles were collected after 48 h and filtered through 0.45 µm pore sterile filters (Corning, NY, USA). Then, 3 × 10^5^ OCI-AML 3 cells were plated in six-well plates (Eppendorf, MI, Italy) with viral particles together with 4 µg/mL of polybrene to increase transduction efficiency. Plates were centrifuged at 1800 rpm for 45′ and then incubated at 37 °C for 75′ in a 5% (*v*/*v*) CO_2_ humidified incubator. After this, cells were washed twice and resuspended with fresh medium. Then, 72 h post-transduction, cells were selected with puromycin 1 µg/mL treatment for a week. Induction of silencing systems was performed by treating cells with 2 µg/mL doxycycline for 72 h; the brightest RFP^+^ cells were sorted with fluorescence-activated single cell sorting (FACS). Silencing efficiency was assessed by WB and quantitative real-time PCR (qPCR).

### 4.3. Western Blotting Analysis

In the case of OCI-AML 3 SCR and shNS, cells were induced with doxycycline for 72 h, whereas for the panel of AML lines, cells were harvested when confluent; for each condition, 3 × 10^6^ cells were collected. Pellets were washed twice with ice-cold phosphate-buffered saline (PBS) solution and proteins were then extracted with a lysis buffer (50 mM Tris/HCl pH 7.6, 150 mM NaCl, 1% NP-40, 0.5% Na-Deoxycholate, 1 mM EDTA pH 8) containing a protease inhibitor cocktail (Sigma-Aldrich, St. Louis, MI, USA), a phosphatase inhibitor cocktail (Roche, Thermo-Fisher, Waltham, MA, USA) and Na_3_VO_4_ (Sigma-Aldrich, St. Louis, MI, USA). Otherwise, to obtain cellular lysates for proteomic analysis, pellets were washed once with medium without serum and three times with ice-cold PBS and then lysed. Equal amounts of total protein lysates were separated through SDS–PAGE electrophoresis and electrotransferred to nitrocellulose membranes for Western blot analysis. Membranes were blocked for 1 h at RT with 5% (*v*/*v*) non-fat dry milk in PBS with 0.1% (*v*/*v*) Tween 20 and incubated overnight with the following primary antibodies: anti-Nucleostemin (E-8) sc-166460 (1:1000), anti-p53 (DO-1) sc-126 (1:1000) (SantaCruz, Dallas, TX, USA), anti-p21 (DCS60) #2946 (1:1000), anti-β-tubulin #2128 (1:1000), anti-GAPDH (D16H11) XP #5174 (1:2000) (Cell Signaling Technology, Danvers, MA, USA) and anti-β-actin (AC-15) #A5441 (1:40,000) (Sigma-Aldrich, St. Louis, MI, USA). After three washes, the membranes were hybridized with horseradish peroxidase-conjugated secondary antibodies (rabbit and mouse; Biorad, Hercules, CA, USA). Detection of signal bands was performed with Clarity Western ECL substrate (#1705061; Biorad, Hercules, CA, USA) or with a SuperSignal West Dura extended duration substrate kit (#34076; Thermo Fisher Scientific). Images of membranes were acquired with a UvitecFire reader (Cambridge, UK) and analysed with Alliance Uvitec software (Cambridge, UK). The original membranes of each blot are reported in Supplementary Figure S5.

### 4.4. Reverse Transcription and Quantitative Real-Time PCR (RT-qPCR)

OCI-AML 3, OCI-AML 2, HL-60 and THP-1 cellular pellets were collected for each condition, after 72 h induction with doxycycline in the case of OCI-AML 3 SCR and shNS, and lysed in QIAzol Lysis Reagent (Qiagen, Hilden, Germany). Total RNA was extracted and quantified. One microgram of RNA was reverse transcribed using a High-Capacity cDNA Reverse Transcription kit (#4368814; Applied Biosystems, Waltham, MA, USA), according to the manufacturer’s instructions. Real-time qPCR was performed with a CFX96 Touch Real-Time PCR Detection system (Biorad, Hercules, CA, USA) using SsoAdvanced Universal SYBR Green supermix (#1725271; Biorad, Hercules, CA, USA), according to manufacturer’s instructions, and DNA was amplified in 96-well plates (Biorad, Hercules, CA, USA). The primers used at a final concentration of 350 nM were:

NS For 5′-GGGAAGATAACCAAGCGTGTG-3′,
NS Rev 5′-CCTCCAAGAAGTTTCCAAAGG-3′;
CDKN1A/p21 For 5′-CGAAGTCAGTTCCTTGTGGAG-3’,
CDKN1A/p21 Rev 5′-CATGGGTTCTGACGGACAT-3’;
TP53/p53 For 5′-AGGCCTTGGAACTCAAGGAT-3’,
TP53/p53 Rev 5′-CCCTTTTTGGACTTCAGGTG-3’;
B2M For 5′-GCTCGCGCTACTCTCTCTTT-3′,
B2M Rev 5′-TGTCGGATGGATGAAACCCA-3′;
β-actin For 5′-CAGCTCACCATGGATGATGATATC-3’,
β-actin Rev 5′-AAGCCGGCCTTGCACAT-3’.

Each sample analysis was performed in triplicate. As a negative control, a sample without a template was used. Cycling parameters consisted of an initial denaturation at 95° for 30 s, followed by 40 cycles of denaturation at 95 °C 15 s and annealing/extension at 57 °C for 30 s. In order to verify the specificity of the amplification, a melting-curve analysis was performed immediately after the amplification protocol. RT-qPCR results were calculated using the ΔΔCt method, with B2M (beta-2-microglobulin) or β-actin used as housekeeping reference genes.

### 4.5. Immunofluorescence and Confocal Imaging Analysis

After 72 h of doxycycline induction, 8 × 10^4^ OCI-AML 3 silenced (shNS) or not (SCR), as well as treated or not, where indicated, were spotted on microscope slides with cytospin centrifugation at 900 rpm for 3 min. Cells were fixed with 4% (*w*/*v*) paraformaldehyde in PBS for 10 min, then permeabilized with 0.1% Triton X-100 in PBS for 10 min and blocked for 1 h with 10% goat serum. At this point, microscope slides were incubated overnight at 4 °C with rabbit anti-p-γH2AX (1:1000; #9718, Cell Signaling Technology, Danvers, MA, USA) properly diluted in blocking solution. The day after, cells were washed three times with PBS 1X and incubated for 1 h with secondary antibody goat anti-rabbit AlexaFluor488 (1:2000; #A32731, Invitrogen, Thermo-Fisher, Waltham, MA, USA) and Hoechst 33342 properly diluted in blocking solution. Cells were washed and then mounted with coverslips. Finally, a ZEISS LSM800 confocal microscope was used for confocal imaging, a 63× numerical aperture lens was chosen to acquire images and a pinhole of 135 µm was used. Images were then analyzed with ZEN blue 3.2 software (ZEISS, Wetzlar, Germany). For DNA foci damage analysis, FoCo software [33] in MatLab R2021b was used.

### 4.6. Cell Viability and Proliferation Assay

After 72 h of doxycycline induction, 2 × 10^5^ OCI-AML 3 cells/well were seeded onto 12-well plates (Eppendorf, MI, Italy). Viable and dead cell counts were performed with Trypan Blue reagent (Sigma-Aldrich, St. Louis, MI, USA) at the indicated time points, considering 72 h post-induction with doxycycline as the starting point. For cell proliferation curves, only viable cells were considered as part of the total number counted for each well considered. The same assays were performed under treatments with different concentrations of etoposide for the time points indicated. For dose–response cell viability assays, 2 × 10^4^ cells per well were seeded onto 96-well plates (Eppendorf, MI, Italy) once incubated with etoposide treatments at indicated concentrations or vehicle solutions at 72 h post-induction with doxycycline, considered as a starting point. At the indicated time points, cells were incubated for 2 h with CellTiter-Blue Cell Viability assay (Promega, Madison, WI, USA), according to manufacturer’s instructions, and then the plates were acquired with the microplate reader Synergy H1 Gen5 (Biotek, Agilent, Santa Clara, CA, USA).

### 4.7. Label-Free Proteomics by Liquid Chromatography Tandem Mass Spectrometry

We performed a shotgun proteomics analysis to evaluate the effect of NS silencing on the OCI-AML 3 cell lines carrying NPM1 mutation A (expressing NPM1c+). Cellular pellets were lysed by sonication in a lysis buffer (urea 6 M in 100 mM Tris/HCl, pH 7.5). After centrifugation of cell debris, the supernatants were tested for protein concentration via Bradford assay (Bio-Rad, Hercules, CA, USA) using Bovine Serum Albumin (BSA, Sigma-Aldrich, St. Louis, MI, USA) standard for the calibration curve in order to digest 50 µg of proteins for each different condition according to the filter-aided sample preparation (FASP) method. For protein label-free identification and quantification, a tryptic digestion was carried out overnight at 37 °C (Merck KGaA, Darmstadt, Germany). Tryptic peptides from each sample were analyzed in triplicate with LC–MS/MS using the UltiMate^TM^ 3000 UPLC (Thermo Fisher Scientific, Waltham, MA, USA) chromatographic system coupled to the Orbitrap Fusion^TM^ Tribrid^TM^ (Thermo Fisher Scientific, Waltham, MA, USA) mass spectrometer, as already reported in our previous work [38]. Moreover, LC–MS/MS analysis was used to further corroborate the NPM1 mutation A (coding for NPM1c+ protein) in OCI-AML 3 cell lines using two fasta files with the two respective sequences of the NPM1 Wild-Type (WT) and NPM1 mutated proteins (see Supplementary Table S1, Figure S6). Indeed, Proteome Discoverer software (Thermo Fisher Scientific, Waltham, MA, USA) was utilized to find the peptides, from the two respective NPM1 sequences, present in the samples OCI-AML 3 SCR and OCI-AML 3 shNS. The mass spectrometry proteomics data have been deposited with the ProteomeXchange Consortium via the PRIDE [39] partner repository with the dataset identifier PXD034012.

### 4.8. Proteomics Data Processing

Proteomics raw data were processed using the Andromeda peptide search engine in a free computational platform, MaxQuant version 1.6.6.0 (Max-Planck Institute for Biochemistry, Martinsried, Germany) to match the spectra against the UniProt FASTA database (released 2018_04, taxonomy Homo Sapiens, 20,874 entries), as already reported in our previous works [40,41]. LFQ Intensity was used to quantify protein abundance in each sample whenever the protein was identified in at least two analytical replicates for each condition.

Bioinformatics analysis was performed with Perseus version 1.6.10.50 (Max-Planck Institute for Biochemistry, Martinsried, Germany). LFQ intensities were log_2_-transformed to facilitate the calculation of protein expression. Variability between the two different cellular treatments was reported as density plot with Pearson correlation (R^2^) values of mean LFQ intensities value-transformed to log_2_ scale (Supplementary Figure S3). The Volcano Plot function was used to identify the differentially regulated proteins by performing a *t*-test with a threshold *p*-value of 0.05. Finally, protein ratios were uploaded for “Core Analysis” through ingenuity pathway analysis (IPA software, Qiagen, Hilden, Germany). IPA is a tool that can statistically map modulated proteins for their functional annotations, such as canonical pathways, upstream regulators and downstream effects networks, through gene ontology and pathway analysis [42]. In this way, it is possible to identify metabolic pathways and secondary genes/proteins inhibited (z-score ≤ −2.00) and/or activated (z-score ≥ 2.00).

### 4.9. Statistical Analysis

Statistical analyses were performed for each type of experiment by using GraphPad Prism (GraphPad software, Inc., La Jolla, CA 92037, USA).

## Figures and Tables

**Table 1:** List of the main differential diseases and biological functions. Activated or inhibited processes in the comparison of OCI-AML 3 shNS/OCI-AML 3 SCR cells. Table reports the predictive z-scores (orange: activation, z-score ≥ 2.00; blue: inhibition, z-score ≤ −2.00). Color is proportional to z-score value. The homologous recombination biological function is highlighted in bold because it was further investigated.

Diseases and Biological Functions	z-Score Activation
Cell death of cancer cells	**3.28**
Bone marrow lesion	**2.10**
Homologous recombination of cells	**−2.15**
Cell survival	**−2.18**
DNA recombination	**−2.25**
**Homologous recombination**	**−2.30**
Cell movement	**−2.48**

**Table 2:** List of the main differential upstream regulators. Activated or inhibited gene regulators in the comparison of OCI-AML 3 shNS/OCI-AML 3 SCR cells. Table reports the predictive z-scores (orange: activation, with significant z-score ≥ 2.00; blue: inhibition, with significant z-score ≤ −2.00). Color is proportional to z-score value. The CDKN1A upstream regulator is highlighted in bold because it was further investigated.

Upstream Regulators	Protein Name	z-Score Activation
*NFE2L2*	Nuclear factor erythroid 2-related factor 2	**−3.29**
*MITF*	Microphthalmia-associated transcription factor	**−3.14**
*MRTFB*	MKL/myocardin-like protein 2	**−3.12**
*CEBPB*	CCAAT/enhancer-binding protein beta	**−2.57**
*ATG7*	Ubiquitin-like modifier-activating enzyme ATG7	**−2.53**
*COPS5*	COP9 signalosome complex subunit 5	**−2.45**
*SYVN1*	E3 ubiquitin-protein ligase synoviolin	**−2.33**
*NFE2L1*	Nuclear factor erythroid 2-related factor 1	**−2.17**
*HSF1*	Heat shock factor protein 1	**−2.08**
*YAP1*	Yes1 associated transcriptional regulator	**−2.08**
*MET*	Hepatocyte growth factor receptor	**−2.01**
*MYC*	Myc proto-oncogene protein	**−2.00**
*HOXA3*	Homeobox protein Hox-A3	**2.00**
*FMR1*	Synaptic functional regulator FMR1	**2.02**
*RBL1*	Retinoblastoma-like protein 1	**2.12**
*HDAC1*	Histone deacetylase 1/2	**2.16**
*EGR1*	Early growth response protein 1	**2.16**
*ZBTB16*	Zinc finger and BTB domain-containing protein 16	**2.16**
***CDKN1A***	Cyclin-dependent kinase inhibitor 1	**2.31**
*RICTOR*	Rapamycin-insensitive companion of mTOR	**2.32**
*SOX11*	Transcription factor SOX-11	**2.36**
*NME1*	Nucleoside diphosphate kinase A	**2.43**
*MXD1*	Max dimerization protein 1	**2.93**

## Data Availability

The data used are available via ProteomeXchange with the identifier PXD034012.

## Supplementary Materials

The following supporting information can be downloaded at: https://www.mdpi.com/article/10.3390/ijms23147655/s1.
