# Supplementary material for: Proteomic Investigation of the Role of Nucleostemin in Nucleophosmin-Mutated OCI-AML 3 Cell Line
Source: Int J Mol Sci. 2022 Jul 11;23(14):7655. doi: 10.3390/ijms23147655 (PMC9317519; doi:10.3390/ijms23147655)

Proteomic Investigation of the Role of Nucleostemin in Nucleophosmin-mutated OCI-AML 3 Cell Line

Ilaria Cela[1,2,†], Maria Concetta Cufaro[2,3,†], Maurine Fucito[1,2], Damiana Pieragostino[1,2], Paola Lanuti[2,4], Michele Sallese[1,2], Piero Del Boccio[2,3], Adele Di Matteo[5], Nerino Allocati[1], Vincenzo De Laurenzi[1,2], Luca Federici[1,2,*]

[1]Department of Innovative Technologies in Medicine & Dentistry, University ''G. d'Annunzio'' of Chieti-Pescara, 66100, Chieti, Italy; ilaria.cela@unich.it (I.C.); maurine.fucito@unich.it (M.F.); damiana.pieragostino@unich.it (D.P.); michele.sallese@unich.it (M.S.); nerino.allocati@unich.it (N.A.); Vincenzo.delaurenzi@unich.it (V.D.L.)
[2]Center for Advanced Studies and Technology (CAST), University "G. d'Annunzio" of Chieti-Pescara, 66100, Chieti, Italy; maria.cufaro@unich.it (M.C.C.); paola.lanuti@unich.it (P.L.); piero.delboccio@unich.it (P.D.B.).
[3]Department of Pharmacy, University ''G. d'Annunzio'' of Chieti-Pescara, 66100, Chieti, Italy
[4]Department of Medicine and Aging Science, University ''G. d'Annunzio'' of Chieti-Pescara, 66100, Chieti, Italy
[5]Institute of Molecular Biology and Pathology, National Research Council of Italy, P.le Aldo Moro 5, 00185, Rome, Italy, adele.dimatteo@cnr.it (A.D.M.).

*Correspondence: Luca Federici, luca.federici@unich.it (L.F.)

† These authors contributed equally to this work

Supplementary Table S1. Protein quantification: List of the quantified proteins in SCR and shNS OCI-AML 3 cell lines. Table reports the raw data, the protein quantification, the fold change and statistical analyses (Volcano Plot) to identify the differential proteins.

Supplementary Table S2. Downstream functions and Upstream Regulators. List of downstream and upstream regulators predicted by IPA software.

Supplementary Table S3. Summary of all peptides found in the different samples and their characteristics. For each peptide found, the corresponding protein sequence is reported together with eventual modifications detected and the range relative to position of the peptide within NPM1 protein sequence.

Confidence	Peptide Sequence	Modifications	Positions in Proteins	Marked		Found in samples	
				NPM1 WT	NPM1 Mut	OCI-AML 3 shNS	OCI-AML 3 SCR
●	[R].MTDQEAIQDLCLAVEEVSLR.[K]	1xCarbamidomethyl [C11]	[278-297]	□	■	■	■
●	[R].MTDQEAIQDLCLAVEEVSLR.[K]	1xCarbamidomethyl [C11]; 1xOxidation [M1]	[278-297]	□	■	■	■
●	[K].DELHIVEAEAMNYEGSPIK.[V]	None	[55-73]	■	■	■	■
●	[K]. DELHIVEAEAMNYEGSPIK.[V]	1xOxidation [M11]	[55-73]	■	■	■	■
●	[K].FINYVK.[N]	None	[268-273]	■	■	■	■
●	[K].GPSSVEDIK.[A]	None	[240-248]	■	■	■	■
●	[K].LAADEDDDDDDEEDDDEDDDDDDFDDEEAEEK.[A]	None	[158-189]	■	■	■	■
●	[K].LAADEDDDDDDEEDDDEDDDDDDFDDEEAEEKAPVK.[K]	None	[158-193]	■	■	■	■
●	[K].LAADEDDDDDDEEDDDEDDDDDDFDDEEAEEKAPVKK.[S]	None	[158-194]	■	■	■	■
●	[K].MSVQPTVSLGGFEITPPVVLR.[L]	None	[81-101]	■	■	■	■
●	[K].MSVQPTVSLGGFEITPPVVLR.[L]	1xOxidation [M1]	[81-101]	■	■	■	■
●	[R].TVSLGAGAKDELHIVEAEAMNYEGSPIK.[V]	None	[46-73]	■	■	■	■
●	[R].TVSLGAGAKDELHIVEAEAMNYEGSPIK.[V]	1xOxidation [M20]	[46-73]	■	■	■	■
●	[K].VDNDENEHQLSLR.[T]	None	[33-45]	■	■	■	■
●	[R].MTDQEAIQDLWQWR.[K]	1xOxidation [M1]	[278-291]	■	□	■	■
●	[R].MTDQEAIQDLWQWR.[K]	None	[278-291]	■	□	■	■

Figure S1: Basal protein levels of Nucleostemin in a panel of AML cell lines. (a) Western blot (WB) images of NS protein levels in a panel of AML cell lines: OCI-AML 3 (AML FAB M4), OCI-AML 2 (AML FAB M4), HL-60 (AML FAB M2), and THP-1 (AML FAB M5). 30 µg of total lysates were loaded for each sample. (b) Histogram represents intensity levels of NS protein bands of WB image normalized for β-actin protein expression. (c) NS mRNA expression levels in AML panel evaluated by qRT-PCR and normalized to actin mRNA expression levels.

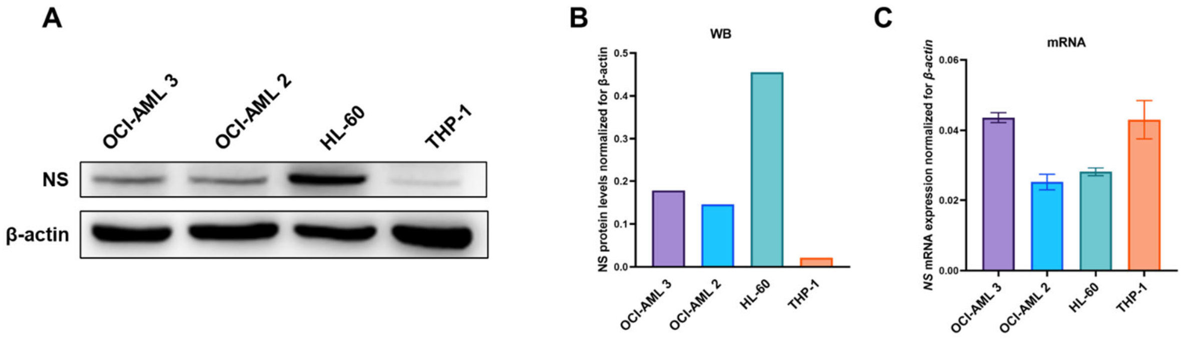

Figure S2: Cell proliferation curves of OCI-AML 3 SCR and shNS. OCI-AML 3 SCR and shNS cells were counted at each time point after 72h of doxycycline treatment to induce NS silencing. Cells were counted by Trypan blue exclusion and expressed as total number of cells. NS, nucleostemin**.**

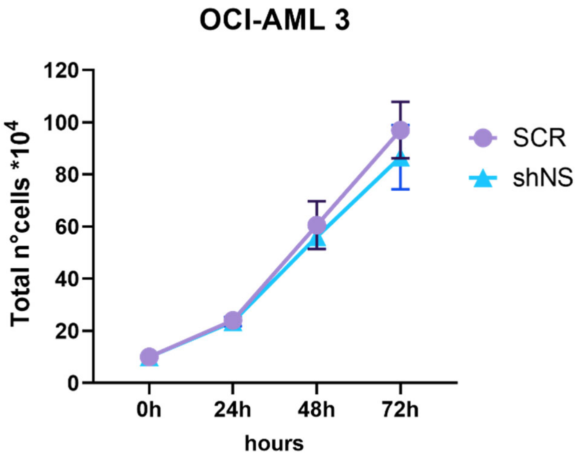

Figure S3: Protein expression correlation of the two different AML cellular models. Protein expression is reported as density value of LFQ Intensity. Data points with the highest density are light blue, on the contrary data points with lowest density are bright green. The color gradient is reported in the legend. In the figure the variability between two different cellular lines is reported as Pearson correlation (R^2) as $\log_2$ LFQ Intensity values.

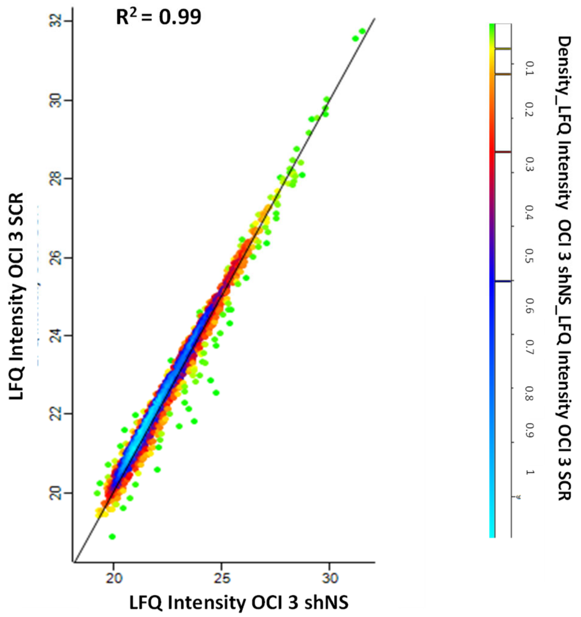

Figure S4: IPA networks legend. The figure shows the color and shape key to read the IPA networks reported in the work.

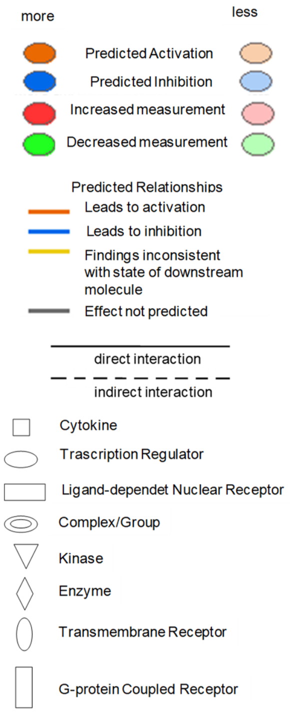

Figure S5: Original membranes of western blot images. (A) NS western blot membranes and its specific primary antibody datasheet, comprised of expected molecular weight. (B) β-tubulin western blot membrane and its specific primary antibody datasheet, comprised of expected molecular weight. (C) CDKN1A/p21 western blot membrane and its specific primary antibody datasheet, comprised of expected molecular weight. (D) TP53/p53 western blot membrane and its specific primary antibody datasheet, comprised of expected molecular weight. (E) GAPDH western blot membranes and its specific primary antibody datasheet, comprised of expected molecular weight. (F) β-actin western blot membrane and its specific primary antibody datasheet, comprised of expected molecular weight.

A

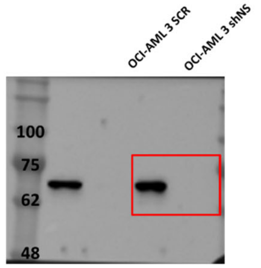

SDS-PAGE 10% - Mw: Sharpmass VI (Euroclone, Italy)

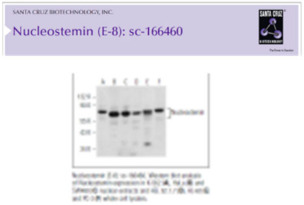

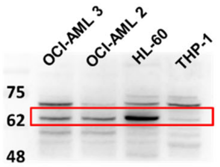

SDS-PAGE 10% - Mw: Sharpmass VI (Euroclone, Italy)

B

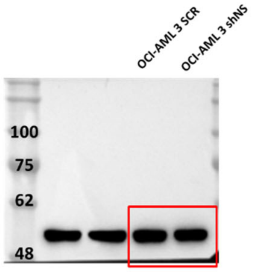

SDS-PAGE 10% - Mw: Sharpmass VI (Euroclone, Italy)

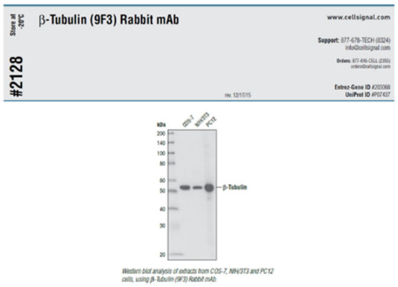

C

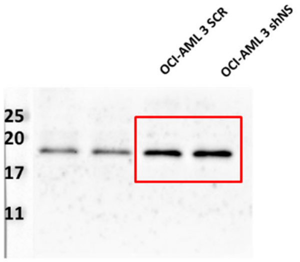

SDS-PAGE 15% - Mw: Sharpmass VI (Euroclone, Italy)

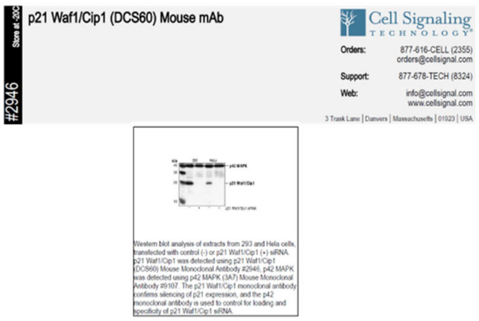

D

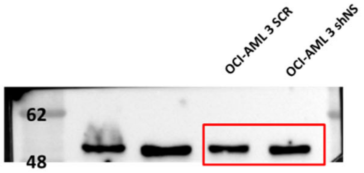

SDS-PAGE 10% - Mw: Sharpmass VI (Euroclone, Italy)

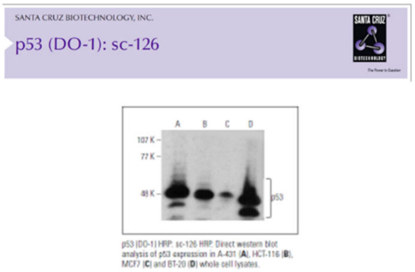

E

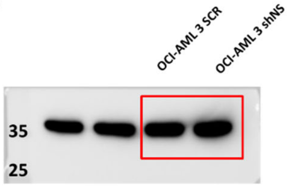

SDS-PAGE 10% - Mw: Sharpmass VI (Euroclone, Italy)

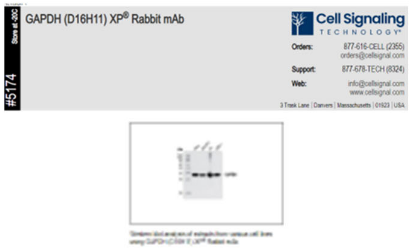

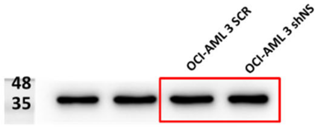

SDS-PAGE 15% - Mw: Sharpmass VI (Euroclone, Italy)

F

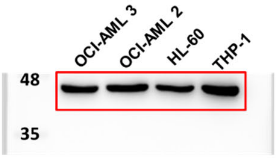

SDS-PAGE 10% - Mw: Sharpmass VI (Euroclone, Italy)

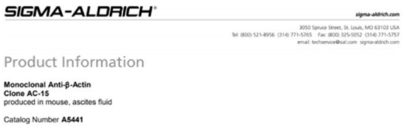

Figure S6. LC-MS/MS analysis of NPM1 mutated protein. Here is emphasized the full sequence of NPM1 Mutated with peptides that allowed to identify the protein found in OCI-AML 3 samples highlighted in green. All peptides found in the different samples are listed in Table S1. Peptides in blue and orange are specific to NPM1 Wild-Type (WT) and NPM1 Mutated (Mut), respectively. Two specific peptides (blue) of the NPM1 Wild-Type were found in OCI-AML 3 validating the presence of NPM1 Wild-Type in OCI-AML 3 SCR and OCI-AML 3 shNS. Moreover, two specific peptides (orange) of the NPM1 Mutated were found in OCI-AML 3 validating the presence of NPM1 Mutated.

Mutation	Protein Sequence
NPM1 Wild-Type	^{286}DLWQWRKSL^{294}
NPM1 Mutated	^{286}DLCLAVEEVSLRK^{298}

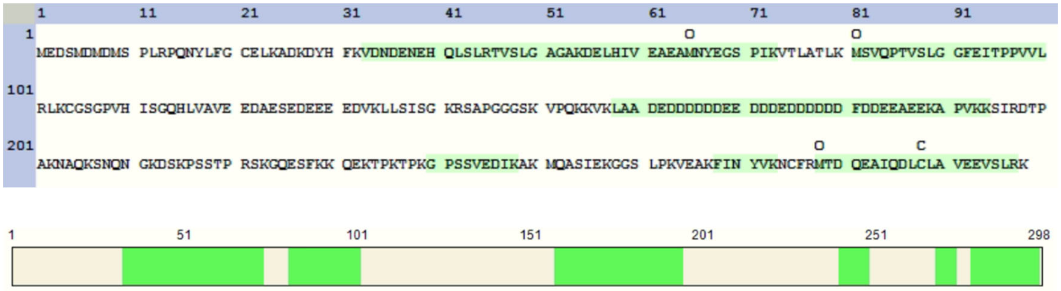

Supplement: Supplementary file 1 [file ijms-23-07655-s001.zip › ijms-1771461-supplementary.pdf]
